# What relatives of older medical patients want us to know - a mixed-methods study

**DOI:** 10.1186/s12912-018-0304-0

**Published:** 2018-07-28

**Authors:** Ditte Maria Sivertsen, Louise Lawson-Smith, Tove Lindhardt

**Affiliations:** 1 Optimed, Clinical Research Centre (Section 056), Copenhagen University Hospital Hvidovre, Kettegård Allé 30, DK-2650 Hvidovre, Denmark; 2 Novo Nordisk A/S, Vandtårnsvej 114, 2860 Søborg, Denmark; 3 Department of Internal Medicine, Copenhagen University Hospital Herlev, Herlev Ringvej 75, 2730 Herlev, Denmark

**Keywords:** Acute hospitalisation, Collaboration, Free text, Older medical patient, Relatives

## Abstract

**Background:**

Relatives of acutely hospitalised older medical patients often act as case managers during a hospital trajectory. Therefore, relatives’ experiences of collaboration with staff and their involvement in care and treatment are highly important. However, it is a field facing many challenges. Greater knowledge of the values and areas that are most important to relatives is needed to facilitate the health care staff to better understand and prepare themselves for collaboration with relatives and to guide family care.

**Methods:**

The aims were to 1) describe the aspects of collaboration with staff during the hospital care trajectory emphasised by relatives of older medical patients 2) compare the characteristics of relatives who wrote free-text notes and those who did not. Relatives of acutely hospitalised older medical patients responded to a structured questionnaire (*n* = 180), and nearly half wrote free-text comments (*n* = 79). Free text was analysed with qualitative content analysis. Differences between (+) free text/ (−) free text groups were analysed with χ^2^ test and Kruskal-Wallis test.

**Results:**

Analysis disclosed three categories I) *The evasive white flock*, concerning the experienced evasiveness in staff attitudes and availability, II) *The absence of care* as perceived by the relatives and III) *Invisible & unrecognised* describing relatives’ experience of staff’s lack of communication, involvement and interactions with relatives especially regarding discharge.

Significant differences were found between relatives who wrote free-text and those who did not regarding satisfaction, trust and having a health care education.

**Conclusions:**

This study provides knowledge of aspects relatives of older medical patients find particularly problematic and, further, of characteristics of relatives using the free-text field. Overall, these relatives were met with evasiveness from staff, an absence of care and felt invisible and unrecognised in the lacking collaboration with staff. Hence, strategies to ensure quality care and systematic involvement of relatives are needed, and the findings in this study may contribute to, and guide, quality improvement of family centered care in acute hospital wards.

## Background

During a hospital stay older medical patients are often accompanied by relatives. These relatives have important knowledge about their older relative, since they are involved in managing their daily life activities [1, 2], and they often feel responsible for the older person’s wellbeing, monitor their professional care and advocate for quality care aimed at increasing the older person’s chances of staying independent [3, 4].

Health-care utilization, mainly inpatient care, increases with age, especially in high-income countries [5]. In Danish medical wards, patients above 65 years old constitute 53% of all admissions [6]. Both national and international policy strategies focus on increasing the involvement of patients and relatives in care and in care decisions to ensure an individualised care trajectory that meets both patients’ and relatives’ expectations [7, 8]. However, there seems to be a gap between policy and practice, since a national survey shows that patients and their relatives in the Capital Region of Denmark feel less involved in their care trajectory than in other regions [9].

Many relatives take on the role as case manager of the hospital trajectory to pursue continuity and high-quality care for the older patient, and their satisfaction with care and treatment is tied to the degree of collaboration with the hospital staff as well as to their reported feelings of guilt and powerlessness [1]. This indicates that relatives have emotional issues related to the hospital context that affect their experience and perceptions. An Australian study explored the immediate needs of relatives of acutely ill older patients through interviews (*n* = 10) and found that *being informed* and *being there* were essential for relatives. However, participants were included at both medical and surgical wards and therefore differs from our patient group of older medical patients [10]. A systematic review from England examining both patients’ and relatives’ perspectives in acute care settings found that relational approaches to care led to more positive experiences during acute hospitalization [11]. As noted, collaboration between relatives and staff is highly relevant when caring for older patients, but several studies show that this can be hard to achieve [4, 12, 13]. As an example, a review of staff-family relationships found that while families of older people value collaboration in care, staff members acknowledge its importance, but have difficulty translating theory into practice [12]. Relatives report that they have to stand up for themselves and for the patients in order to overcome these conflicts in values and the discrepancies in defining the patient’s situation [3, 14]. Greater knowledge of the values and areas that are most important to relatives would help the healthcare staff better understand and prepare themselves for collaboration with relatives.

Asking respondents to add free-text comments in questionnaires is common practice; However, it is less common to use them for analysis. Yet, they may increase our understanding of respondents’ responses and experiences and identify areas that are particularly important to the target group. This may guide development of clinical practice as well as future research [15]. An unexpected large amount of questionnaires were returned with free-text notes, and that raised our interest in what they wanted to tell us, as well as in what characterises the respondent, who puts time and effort into making free-text notes in an already comprehensive and demanding questionnaire. Out of respect for this effort, we further found that we had an obligation to use these data. The data material is part of a bigger study, and the quantitative questionnaire results are presented elsewhere [16]. To our knowledge, no studies have, until now, analysed free-text comments from relatives of older acutely admitted medical patients.

## Methods

### Aim

The aim of this study was to explore the use and content of a free text possibility in a structured survey by:

- Describing the aspects of collaboration with staff during the hospital trajectory emphasised by relatives of older medical patients.
- Comparing characteristics of relatives who wrote free-text notes and those who did not.

### Design

A cross-sectional, descriptive and comparative mixed-method design was applied analysing free text data from a structured survey study.

### Setting and data collection

The study was conducted at the Medical Department of a Copenhagen University Hospital covering seven wards. Patients matching inclusion criteria were approached consecutively after admission, informed about the project and asked for permission to contact the relative that helped them the most. If patients gave written consent, the relative was contacted by phone to obtain verbal permission to send the questionnaire to them. An envelope containing the questionnaire, written information about the project and a prepaid return envelope was then sent to the relative. Returning the questionnaire was considered as written consent according to Danish law practice. Questionnaires were completed after the patients’ discharge. Data collection took place from November 2010–November 2011.

### Participants

Relatives of older medical patients (≥65 years, acutely admitted, living at home, able to cooperate, and having comorbidities or receiving home care) were eligible for inclusion. *Relatives* were the persons appointed by the patient as the one providing the most help; this could be a family member, friend or acquaintance.

### The family collaboration scale

The Family Collaboration Scale (FCS) is a validated structured questionnaire measuring collaboration with health care professionals as experienced by relatives of older patients [17]. FCS covers four dimensions: 1) Attributes of collaboration; 2) Prerequisites of collaboration; 3) Outcomes of collaboration; and 4) Promoters of and barriers to collaboration. A free-text field on the last page encourages subjective descriptions of experiences and reflections: “*If you have any additions regarding the collaboration with staff that you think the questionnaire did not deal with sufficiently, feel free to write them here*”.

### Data analysis

#### Quantitative data

Respondents who provided written comments were compared with those who did not in terms of age, gender, kinship, education, helping frequency, duration of caregiving and scores of trust and satisfaction with the hospital trajectory in the structured part of the FCS. To give an overall picture of the comments a frequency count was performed to identify the ratio of positive (i.e. praise) and critical comments. This was done by counting the predominance of sentences with positive respectively critical content. Categorical data were analysed with χ^2^ test, and Kruskal-Wallis test was used to analyse numerical data. SAS 9.3 software was used for statistical analysis.

#### Qualitative data

The handwritten comments were transcribed and merged into one document while keeping the ID number of each comment for identification. Qualitative content analysis was performed [18]. The data set was read several times to achieve a general understanding. Hereafter the text was divided into *meaning units*, which were further reduced into *condensed meaning units*. A *code* was derived representing the core of each meaning unit. Codes were sorted into categories, which were labelled in accordance with the meaning content (see Table 1). Two of the authors (DMS, TL) performed this analysis and discussed the findings until reaching consensus. The authors’ pre-understandings will influence the analysis and interpretation of data; hence the two authors strived to be aware of their pre-understandings and challenge each other in the analysis process.

**Table 1:** Example of the analytical process used for qualitative content analysis

Meaning unit	Condensed meaning unit	Code	Category
*Most of them [staff] were stressed and had very little time to inform relatives when asked* (Daughter, age 43)	Staff was stressed and gave little information	Workload is a barrier for communication	The evasive white flock
*I find it very difficult to tell the difference between nurses, doctors, porters* etc. *it makes it very difficult to approach the right one – in my case a nurse* (Son, age 36)	Difficult to distinguish between staff groups	Approachability	The evasive white flock
*Even though I made staff aware that my father lived on nutritional protein drinks after surgery for throat cancer, they kept serving him brown bread and stuff like that. For a whole day he got nothing to eat or drink…* (Daughter, age 65)	Staff not considerate of eating issues and did not provide appropriate food	Basic care need: Eat and drink adequately	Absence of care
*Came home in rainy weather in a taxi wearing nothing but slippers, white long underpants and an undershirt. It was cold.* (Son, age 66)	Patient was sent home in his underwear in cold weather	Basic care need: maintaining body temperature and dignity	Absence of care
*We had to seek all information ourselves, and a discharge meeting was held only after I put my foot down* (Son, age 59)	Information and involvement only happened upon relative’s own initiative	Lack of communication and involvement	Invisible & unrecognised
*My father was for a while treated as a diabetic patient with insulin injections, although he is not diabetic. I made staff aware of this, but I was rejected. 3 days went by, before they stopped the injections.* (Daughter, age 56)	Staff did not pay attention to the relative, and treated the patient incorrectly	Lack of communication and involvement	Invisible & unrecognised

### Ethical considerations

Written and oral information was given to both patients and relatives, emphasizing that participation was voluntary, that withdrawal from the project could be done at any time and that participants’ identities would be kept confidential. None of the researchers were employed in the participating wards.

## Results

### Participants

We received 180 of the 279 questionnaires that were sent out (response rate, 64.5%). Of these, 79 (44%) had free-text comments. The comments ranged in length from 5 to 1298 words, with a total of 7662 words and a mean of 97 words per comment. Sixty-eight percent of the comments were written by women (see Table 2). There were significant differences between relatives who wrote comments and those who did not. Of those who provided free-text only 38.4% reported high satisfaction at admission, whereas the no-comment group reported 57.6% (*p* = 0.008). The results were similar for the two groups’ satisfaction during hospital stay (41.1% vs. 56.1%, *p* = 0.001) and at discharge (32.9% vs. 43.9%, *p* = 0.030). Also, more respondents who scored low on trust in the structured part of the FCS made notes in the free-text field (39% vs. 54.6%, p = 0.008) and finally those who had a health education elaborated more in free-text (26% vs. 13.3%, *p* = 0.033). There were no significant differences in other background variables between the two groups. However, although not significant, more respondents with high school or university education made free-text comments than did those with public school education.

**Table 2:** Characteristics of relatives who did or did not add free-text comments to the Family Collaboration Scale questionnaire

	Added comments (*n* = 79)	n	No comments (*n* = 101)	n	*P*-value*
Age, years, mean	60.3	78	60.8	98	0.998
Sex, Female, %	67.5	77	66.0	100	0.830
Relationship with patient, %					
Spouse	22.8	79	27.7	101	0.749
Offspring	62.0	79	58.4	101	
Other	15.2	79	13.9	101	
Education, %					
Public school	64.1	78	74.3	101	0.143
High school/ University	35.9	78	25.7	101	
Health education, %	26.0	77	13.3	98	**0.033**
High degree of satisfaction with hospital care**, %					
At admission	38.4	73	57.6	99	**0.008**
During the hospital stay	41.1	73	56.1	98	**0.001**
At discharge	32.9	73	43.9	98	**0.030**
High degree of trust in the provided care**, %					
I trusted that my relative got the care s/he needed	39.0	77	54.6	99	**0.008**

^*^SAS 9.3 was used for the statistical analysis. The χ^2^ test was used to analyze categorical data, and the Kruskal-Wallis test was used to analyze numerical data. *P*-values < 0.05 were considered significant and are highlighted in bold font

**Response categories in the questionnaire were: high degree, some degree, less degree, not at all

Five comments were entirely positive expressing praise and satisfaction with the hospital trajectory, 40 comments were entirely critical and 34 comments were mixed. Overall, the positive statements tended to be more general in nature, while the critical ones tended to provide more detailed descriptions.

### Findings

The encounter with the hospital system was the overarching theme, and communication seemed to be central in all categories. Three categories emerged: *The evasive white flock* describing perceptions of staff attitudes and availability; *The absence of care* describing relatives’ perceptions of hospital care; *Invisible & unrecognised* concerning the lack of involvement and collaboration experienced by relatives. The categories were closely interrelated and had in common a predominant sense of ‘something’ that was missing. We found all of the following categories covered in the questionnaire, hence, these aspects were seemingly either particularly central to relatives, or they were aspects of the trajectory that were particularly poorly handled by the staff, leading to a need in relatives to elaborate further in writing.

#### The evasive white flock

Staff attitudes and availability was a central issue for relatives in their encounter with the hospital system. Staff approachability was also an issue, and relatives described observing the staff and trying to find an appropriate moment to approach them. Several comments included descriptions of a futile search for someone to talk to among the staff. The staff did not appear to be visible and available, creating an unwelcoming atmosphere for the relatives. The search for a relevant person to contact was like a game of hide and seek and several comments described unfriendly behaviour from the staff. Although some were accessible and helpful, others had dismissive attitudes when relatives approached them with questions.

It was quite impossible to speak to a nurse; they were just simply not there, no matter where we searched. When we found one, they did not have the time – “ask someone else” or “he belongs to some other nurse” (…) In some cases the nurses were quite rude when we tried to get information (Daughter, age 64).

The nurses’ unavailability seemed to be a barrier to interaction. Moreover, relatives found it difficult to identify which staff to approach for information. The uniforms all looked the same to the relatives, and the staff was seen as one big group of people, all dressed in white. The text indicated that relatives experienced the ward as a busy environment in which the staff was always short of time and where it was hard for relatives to identify the right person to approach for information about the care of their hospitalized relative. Many relatives reported that the staff seemed busy and stressed. At times this created barriers in the communication process and made relatives hesitate to approach the staff.

The interaction between staff and relatives could be improved if you did not feel like you were interrupting the staff’s routines (…) It is difficult to find the right time to discuss your relative’s situation with the staff without feeling a bit “in the way” (Daughter, age 55).

The relatives felt that they disturbed the staff and interrupted their routines when they wished to talk about the patient’s situation. The working conditions and time pressure were in some comments used as explaining factors for missing interaction, information and staff being dismissive.

The staff in the ward was very busy, but in spite of that, as a relative, it was frustrating that there was no proper communication, one could only feel sorry for the staff having the burden they clearly experienced. However, I am sad that I never felt that I could get a satisfactory answer when I approached them (Granddaughter, age 34).

The comments expressed ambivalent feelings about the staff. Although relatives described being frustrated over poor communication and the lack of satisfactory answers, they also expressed understanding for the staff’s working conditions and used these conditions as explanations for unfortunate events, praising their efforts in a high-pressure working environment.

#### The absence of care

This category describes experiences connected to nursing care. Descriptions of basic care needs that were not met were plenty and regardless of their sympathy for the staff’s workload and time pressure, the relatives expressed strong concerns over patient needs that were neglected. The comments included descriptions such as lack of sufficient nutrition, insufficient personal hygiene, dirty linen and clothing and a general lack of professional and compassionate care.

One relative considered the absence of care to be a contributing factor to the death of his mother and generalized the experience by expressing concern for other patients as well.

My mother was sent home without proper clothing, in just underwear and a thin dressing gown in a transport car without assistance. She had to climb up the stairs to the 1st floor. She was hospitalised again the following day, and she died three days later. I hope that other elderly people are not treated in this manner (Son, age 68).

Comments about drug administration raised issues such as patients not receiving sufficient analgesics, medicine given in the wrong dose, adverse drug events and missing medication reconciliation. Medicine-related errors related to lack of communication were described by relatives who had provided the staff with important information about the medication; however, the staff did not seem to be responsive or to take the information into consideration.

As a daughter, I know all about my mother and her medication, and yet they would not listen, so it [medication] was given incorrectly - disgraceful (Daughter, age 59).

The physical environment and especially the lack of a calm, health-promoting environment, seemed to affect the relatives’ perceptions of hospital care. Several relatives complained about insufficient cleaning of the wards, which gave them a bad impression of the hospital as a whole. Privacy and dignity issues were also a concern. Some perceived sharing a room to reduce dignity and to affect tranquillity and sleep. Loss of dignity was also described when patients were moved, like pieces of furniture, from one room to another several times during their stay.

#### Invisible & unrecognised

Analysis indicated that relatives felt invisible and unrecognised in situations where they actually tried to obtain some kind of collaboration with staff. Their expertise was seemingly not requested, and they were not involved at moments where they considered their participation to be crucial, for instance when getting closer to a date of discharge. Information, coordination and involvement were, in the eyes of the relatives, important for a satisfactory discharge process. Extra home care, medicine or rehabilitation were mentioned as interventions relatives needed to collaborate with staff about. Not being informed or involved in planning or decision processes frustrated the relatives, who described how they had to be proactive and persistent in order to get involved and to obtain adequate information. In some cases, missing information even affected the relationship between the patient and his or her relative, and clear and direct information about the patient’s status was wanted.

My mother died during hospitalisation, and I would like to have had specific information about how serious the situation was. Instead, doctors and nursing staff used general terms and hints (Son, age 55).

Lack of information had consequences for the relatives’ possibility for providing support and planning of the future care. Several relatives reported that information about discharge was not given until a few hours before discharge. This prevented relatives in preparing the patient’s homecoming. Some were not informed of the discharge at all.

Were never informed of the discharge. Were never involved in the process (…) Very poor communication (Daughter, age 45).

Information about how to care for the patient at home was also called for; some relatives expressed uncertainty about which symptoms they should be aware of or how to react if symptoms worsened.

In some cases, patients seemed to be sent home too soon and were, in the relative’s opinion, not ready for discharge. Different kinds of barriers to continuity in the care trajectory were described, e.g. insufficient transition of information through the system and to municipal care agencies, and lack of communication between in-hospital units and other hospitals, as well as between staff and relatives.

The relatives expressed frustration over hospital staff’s lack of interest and sense of responsibility for patient after discharge. Sometimes the relatives contacted the discharging hospital unit for some piece of information or follow-up on the discharge arrangements and were met with indifference.

…Then I called the hospital unit the day after discharge, and I was told that the case was filed now that my mother-in-law was discharged (Daughter-in-law, age 59).

In contrast, The Supported Discharge Team was highlighted as a positive and successful initiative that involved relatives in the discharge process. This team was perceived as being empathic and taking the time to understand the patient in his/her daily context.

## Discussion

The main aim of this study was to explore aspects of the hospital trajectory, relatives needed to emphasize. The three categories that emerged were: the evasive white flock, absence of care and invisible & unrecognised. Relatives needed guidance and, at the same time, felt that they were in the way and a disturbance to the staff. The first category concerning evasiveness of the nurses seemed central to this paradox. Studies of how nurses view collaborations with relatives have found that although relatives are considered an important resource, in practice nurses try to avoid relatives, particularly if they are perceived to be demanding [13, 19]. Lindhardt et al. [13] found a pattern of ‘escape-avoidance’ conduct, which, from the relative’s point of view, may be perceived as unavailability as described in our study. The nurses’ non-verbal communication of time pressure further inhibited communication by making relatives hesitate to approach them to avoid disturbing their work activities. The inaccessibility of nurses and relatives’ reluctance to disturb staff are well-known problems in the collaboration between relatives and nurses. Literature has described these problems in different contexts, e.g. in medical wards [4], nursing home [20] and in complaints from both patients and relatives regarding encounters and communication at a large Swedish hospital [21] indicating its persistence and widespread occurrence. Our results indicated that what researchers have found to be culturally embedded behaviour of nurses is perceived by relatives as inaccessibility and as a barrier to contact and communication.

Absense of care was identified as the second category. Care is the essence of nursing [22]. However, the relatives in our study reported that in their experience, care was not always prioritised in everyday nursing practice, and they described in detail examples of this. There seemed to be a discrepancy between their expectations and the practice they encountered in the acute hospital context. Other studies have shown that relatives of older patients in acute hospital wards provide informal care [23]. Given the inclusion criteria (i.e. comorbidities and receiving home care) it may well be that the relatives in our study were informal caregivers. If so, they may have had special knowledge of the care needed by their hospitalised older relative, and when they observed that these needs were not met by the formal caregivers, it led to frustration. Relatives with a health education more often made comments. Taverner et al. [24] analysed the experiences of registered nurses who were also family caregivers of hospitalised older people, and found that these subjects experienced a culture of care where neglect were normalised, and therefore had to act “*vigil by the bedside*”, causing feelings of distress and disjuncture between their own identity as a nurse and the care they witnessed. Similarly, this vigilant monitoring has been described elsewhere, when caregivers’ unmet expectations were replaced by uncertainty and suspiciousness [4]. Theories of informal caregiving has identified *worry* and *the protective dimension* as central aspects [25, 26]. Studies have shown that some relatives in acute medical wards ‘stand guard’ to protect the patient from flaws and poor care and that they feel responsible for the patient’s wellbeing [14]. The perceived absence of care, in our study, may create such worries and awareness, and this may explain the frustrations and the emphasis on the lack of care and collaboration expressed in the study.

In our study the relatives’ inclination to write free-text comments was highest among those with negative experiences. This tendency is also seen in a large study of patient satisfaction surveys (*n* = 75.769) where the least satisfied patients were most keen to elaborate in free-text [27]. The same applies to Garcia et al. [15] who has examined the use of free-text comments, and concludes that those who comment are either the articulate ones or those who have something negative to elaborate. However, this did not apply for a survey conducted among relatives of hospice patients, which showed that positive comments accounted for 75% of the free text comments [28]; hence, context seemingly is an influential factor. In the characteristics of the participants, we found that relatives scoring low on trust in our study more often elaborated in free-text writing. We cannot tell if these relatives lacked trust from the beginning and therefore were more observant and critical, or if trust disappeared due to the flaws in the care they experienced. However, trust is a value that lies within the concept of caring [29] and has been found to be central in a relative’s collaboration with health care professionals [17]. Relatives hand over their loved ones to the care of hospital professionals, and for informal caregivers, this requires trust. Relatives monitor how this responsibility is handled by the professionals, and whether care is provided with engagement and empathy is likely to form the basis for trust or distrust. In the psychometric testing of the FCS, trust was found to be a special factor dimension, indicating its significance in the nurse-relative collaboration [17]. The trust dimension was shown to be particularly important in the admission phase and to correlate with the quality of contact with nurses, indicating that relational and communicative aspects are related to trust. Further, the physical environment was correlated with trust [17]. The physical environment was mentioned in our study and in conjunction with the evasive nurses, it impaired contact and communication and therefore possibly also trust. Further, the relatives in our study expressed frustration when their need for information was not acknowledged by the staff. Also, if a relative’s knowledge about a patient’s situation is not taken into account, insecurity may develop and trust may be threatened. Studies have pointed out that a lack of care and information creates worries, doubt and distrust [10, 21], and other studies suggest that an accessible, listening and empathic nurse is a prerequisite for successful collaboration with relatives [12, 30].

Closely connected to the experienced evasiveness from the staff was the feeling of being “invisible and unrecognised” in the third category. The lack of exchange of information between relatives and staff stood out in the comments. Relatives, particularly informal caregivers, are important sources of information, with a special need for information, as they often take over the patient’s care after discharge. Communication is a prerequisite for collaboration which again is a prerequisite for sufficient exchange of information between staff and relatives [31]. A Danish study found that poor collaboration was significantly associated with relatives’ low satisfaction with the care trajectory [1]. In accordance with this finding, the majority of the respondents in our study were dissatisfied with the care trajectory, and a central complaint was the lack of collaboration and communication between relatives and nurses. Communication seemed negatively affected by several factors. The relatives described how communication with staff happened when they initiated it, which is in accordance with other studies [32]. This means that seemingly even resourceful relatives, such as our respondents, were unable to obtain the communication and information they needed. Further, our study indicated that discharge was an important time at which the need for coordination and communication was crucial. However, the relatives felt ignored and that their knowledge was not granted. Studies of strategies to improve discharge planning and increase satisfaction, emphasizes an individualised approach where involvement, support and communication are important factors [33, 34].

Seemingly, relatives call for nursing delivered in accordance with nursing values, but nurses seem reluctant to provide it. However, nurses report feelings of guilt and frustration because of their inability to provide good patient care in accordance with their own professional ideals [35]. It is noticeable, that although frustrated and worried by the absence of care and evasive nurses, the relatives in our study saw nurses as victims and sympathised with them due to their stressful working conditions. This, in accordance with the study of Lindhardt et al. [14], in which relatives blamed the system rather than the people working in it. Several studies have described the dilemma of today’s nurses working conditions, where nursing values compete with more powerful, organisational, value systems [31, 36]. New Public Management (NPM) and its value system governs the public sector and eldercare in Nordic countries including Denmark [37]. It represents an administrative-economic rationale and stands in contrast to nurses’ professional medical rationale [36]**.** Effectiveness and productivity are central values in NPM and form the fundamental conditions for clinical practice in which nurses are supposed to provide care, and the nursing values may therefore be challenged within this context.

### Strengths and limitations

Our results disclose aspects seemingly of particular importance to the participants in the survey, since the questionnaire had already dealt with these issues, and yet the respondents felt the need to elaborate further after completing the structured questionnaire. This provides us with information that may be used in quality improvement efforts and when planning collaborative interventions targeted relatives.

There is, however, a risk of a biased sample for several reasons. Firstly, it takes a certain amount of mental strength and energy to add notes to an already extensive structured questionnaire. Potentially, those who did not add free text were the ones under most strain. Secondly, more dissatisfied relatives added free text notes, and the notes were more often critical, a tendency described elsewhere [15, 27]. Thirdly, relatives with a health education more often wrote comments. They may have professionally-based expectations to the care trajectory, be more likely to notice flaws and may possibly be more willing to return the questionnaire. Hence, the sample was not representative, and this limits the generalizability of the conclusion.

This study demonstrated the value of combining qualitative and quantitative elements, since analysis of the survey data offered information both about the issues relatives found especially important and therefore needed to emphasize and the characteristics of respondents with particular need to elaborate in free text.

### Implications for clinical practice

The free-text comments analysed in this study indicated that quality of care for older patient varies and that active strategies to ensure quality care and involvement of relatives are needed. Nursing managers should provide a framework and conditions for structured involvement in clinical practice at the cultural-, educational- and organisational levels. The perceived unavailability of nurses should be addressed by nursing leaders and clinical managers, who should encourage and facilitate constructive interactions and collaborations with relatives. There is a need to analyse nursing workloads and to prioritise nursing care. Working systematically with feedback meetings and user panels to analyse individual cases and organisational in-ward developments will ensure that valuable observations and knowledge of patients and relatives are considered. Relatives are clearly allies for nurses: they are motivated to provide good care while having sympathy and understanding for the staff’s high-pressure environment. Including relatives in the planning and providing of care may promote nursing core values in clinical settings, increase the quality of care for the patient and the satisfaction among relatives.

## Conclusions

In line with other studies investigating the experiences of relatives at hospitals, relatives in our study reported feeling uninvolved and ‘in the way’, which was a barrier for contact and communication with staff. Furthermore, collaboration was inhibited by the nurses’ evasiveness. Experiences with low-quality care seemingly sparked the inclination to write free-text comments, as we identified significant differences regarding the satisfaction and trust scored by relatives who wrote free-text comments versus those who did not. Factors such as lack of contact with staff, absence of information and care and not being involved were frustrating to relatives, who seemed to be keen observers of the busy atmosphere of acute wards and to have a clear vision of good quality care. Further studies are needed to investigate characteristics of relatives who want to collaborate with staff and to test interventions in acute care settings aimed at systematically involving relatives.

## Abbreviations

FCS: The Family Collaboration Scale
NPM: New Public Management
